# Administering an appeasing substance to beef calves at weaning to optimize welfare and productivity

**DOI:** 10.1093/tas/txaa101

**Published:** 2020-12-22

**Authors:** Kelsey M Schubach, Reinaldo F Cooke, Alice P Brandão, Bruna Rett, Vitor Ferreira, Giovanna Scatolin, Eduardo A Colombo, Courtney L Daigle, Ky G Pohler, Bruno I Cappellozza

**Affiliations:** 1 Department of Animal Science, Texas A&M University, College Station, TX; 2 Faculdade de Medicina Veterinária e Zootecnia, Universidade Estadual Paulista, Botucatu, Brazil; 3 ║Nutricorp, Araras, Brazil

## INTRODUCTION

Beef cattle are inevitably exposed to psychologic, physiologic, and physical stressors associated with routine management throughout their productive lives (Cooke, 2017). These include the stress caused by weaning, which stimulates adrenocortical and acute-phase protein responses that have immediate and long-term impacts on calf growth and immunity (Carroll and Forsberg, 2007). Hence, strategies to alleviate the stress elicited by weaning are warranted to promote calf performance and welfare in cow–calf and subsequent feeding operations.

One strategy to enhance overall cattle welfare and productivity is the use of appeasing pheromones, which have been isolated in multiple species. In cattle, the synthetic analogue of the appeasing pheromone is based on a mixture of fatty acids, reproducing the composition of the natural substance (Cooke et al., 2020). The synthetic analogue (bovine appeasing substance, BAS) is expected to have calming effects in cattle, improving welfare and productivity upon stressful management procedures. Previous research demonstrated that administration of BAS to beef calves upon weaning alleviates the resultant acute-phase protein reaction and improved growth during a 45-d postweaning period (Cooke et al., 2020). However, research investigating the effects of BAS use in beef cattle is still limited and warranted to further understand its biological and productive benefits. We hypothesized that administration of BAS to beef calves upon weaning will alleviate adrenocortical and acute-phase protein responses and improve feed intake and efficiency, resulting in improved performance during a 42-d postweaning period. This experiment evaluated the impacts of BAS administration at weaning on behavioral, performance, and physiological responses of beef calves.

## MATERIALS AND METHODS

This experiment was conducted at the Texas A&M—Beef Cattle Systems (College Station, TX). Animals were cared for in accordance with acceptable practices and experimental protocols reviewed and approved by the Texas A&M—Agriculture Animal Care and Use Committee (#2019-019A).

### Animals and Treatments

Eighty calves (¾ Angus x ¼ *Bos indicus*; 40 heifers and 40 steers) were used in this experiment. At weaning (day 0), calves were ranked by sex, body weight (BW), age (233 ± 2 d), and initial temperament (Cooke, 2014), and assigned to receive BAS (Nutricorp; *n* = 40) or placebo (diethylene glycol monoethyl ether; CON; *n* = 40). Calves were immediately segregated by treatment into one of two groups and processed again for treatment administration. Treatments (5 mL) were applied topically to the nuchal skin area of each animal (Cooke et al., 2020). Upon segregation, treatment groups had no physical contact, and calves within each treatment group were ranked by the aforementioned variables and allocated to one of eight drylot pens (10 calves per pen; four pens per treatment) with an empty pen maintained between pens of differing treatments to preserve distance.

Calves were vaccinated against *Clostridium* (Covexin 8; Merck Animal Health, Omaha, NE) and respiratory pathogens (Titanium 5; Elanco Animal Health, Greenfield, IN), and administered an anthelmintic (Dectomax; Zoetis) on day 0. Calves had free choice access to water and a total-mixed ration (TMR) during the experiment (31.8% ground corn, 30.0% dried distillers’ grains, 28.8% alfalfa hay, 7.0% liquid molasses, and 2.4% mineral mix; as-fed basis). The TMR was offered once daily (0800 h), in a manner to yield 10% residual orts.

### Sampling and Laboratorial Analysis

Full BW was recorded on days 0, 3, 7, 14, 21, 28, 35, 42, and 43, and individual average daily gain (ADG) calculated by modeling linear regression of BW against sampling days. Intake of TMR (dry matter basis) from each pen was evaluated daily, divided by the number of calves within each pen and expressed as kg per calf per day. Total BW gain and TMR intake of each pen were used for feed efficiency (G:F) calculations.

On day 0, each calf was fitted with a pedometer (HJ-321; Omron Healthcare, Inc., Bannockurn, IL) placed inside a polyester patch fixed behind their right shoulder to assess physical activity. Pedometer results were recorded when calves were processed for sampling events as in Schubach et al. (2017). Chute score and exit velocity were assessed (Cooke, 2014), and blood samples collected via jugular venipuncture concurrently on days 0, 3, 7, 14, 21, 28, 35, and 42. Blood samples were placed immediately on ice, centrifuged (2,500 × *g* for 30 min; 4 °C) for plasma harvest, and stored at −80 °C. Hair samples were collected from the tail-switch of all calves on days 0, 14, 28, and 42 and stored at −80 °C (Schubach et al., 2017). Plasma samples were analyzed for haptoglobin and cortisol, and hair samples for cortisol as in Colombo et al. (2019). Across all assays, the intra and interassay coefficient of variation were ≤8.4%.

### Statistical Analysis

Calf was considered the experimental unit for all analyses, and data were analyzed using the MIXED procedure of SAS (SAS Inst. Inc., Cary, NC). All data were analyzed using calf(pen × treatment × sex) and pen(treatment) as random variables, but for TMR intake and G:F that used pen(treatment) as the random variable. Model statements contained the fixed effects of treatment, day, sex, and all resultant interactions. Plasma and hair variables were analyzed using results from day 0 as an independent covariate. The specified term for all repeated statements was day, with pen(treatment) as subject for TMR intake and calf(pen × treatment × sex) as subject for all other analyses. The covariance structure used was first-order autoregressive, which provided the smallest Akaike information criterion. Results are reported as least square means, or covariately adjusted least square means for plasma and hair variables, and separated using least square differences. Significance was set at *P* ≤ 0.05 and tendencies at *P* > 0.05 and ≤ 0.10. Results are reported according to main effects if no interactions were significant or according to the highest order interaction detected.

## RESULTS

**Figure 1.**
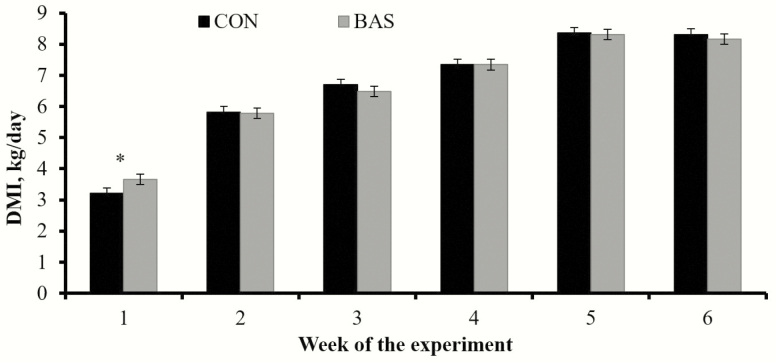
Feed intake on a weekly basis of beef calves receiving (BAS; *n* = 40) or not (CON; *n* = 40) a bovine appeasing substance at weaning (day 0). A tendency for treatment × week interaction was detected (*P =* 0.08). Within days: *P = 0.05.

Average daily gain and final BW (day 42) did not differ (*P* ≥ 0.52) between treatments (Table 1). Nonetheless, ADG from days 0 to 28 was greater (*P* = 0.05) in BAS vs. CON calves (0.96 vs. 0.80 kg/d, respectively; SEM = 0.05). No treatment effects were detected (*P* = 0.94) for daily TMR intake (Table 1). When evaluated on a weekly basis, TMR intake was greater (*P* = 0.05) during the first week for BAS vs. CON calves and similar (*P ≥* 0.44) for weeks 2 to 6 (Figure 1). No treatment effects were detected (*P* ≥ 0.39) for overall G:F (Table 1), or G:F evaluated on a weekly basis (data not shown). It should be noted that no incidence of morbidity or mortality was observed during the experiment.

**Table 1.** Performance variables of beef calves receiving (BAS; *n* = 40) or not (CON; *n* = 40) a bovine appeasing substance at weaning (day 0)^1^

Item	CON	BAS	SEM	*P*-value
Growth parameters				
Initial body weight (day 0), kg	184.8	185.1	3.7	0.95
Final body weight (day 42), kg	228.4	230.6	4.2	0.71
Average daily gain, kg/d	1.15	1.21	0.05	0.52
Feed intake, kg/d	6.43	6.45	0.10	0.94
Feed efficiency, g/kg	157	165	6.7	0.45

^1^Feed intake was recorded daily from days 0 to 42 by measuring offer and refusals from each pen, divided by the number of calves within each pen, and expressed as kg per calf/d. Feed efficiency was calculated using total feed intake days 0 to 42, and body weight gain of each pen from days 0 to 42. Average daily gain calculated by modeling linear regression of body weight against sampling days (0, 3, 7, 14, 21, 28, 35, 42).

**Figure 2.**
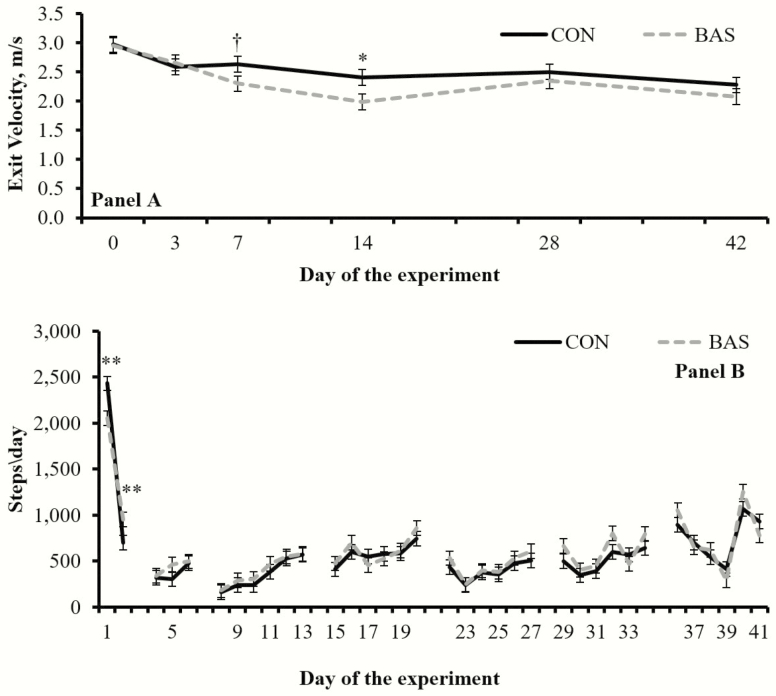
Exit velocity (A; as in Cooke, 2014) and physical activity (B) of beef calves receiving (BAS; *n* = 40) or not (CON; *n* = 40) a bovine appeasing substance at weaning (day 0). Treatment × day interactions were detected for both variables (*P* ≤ 0.04). Within days: ^†^*P* = 0.08; *P = 0.03; **P < 0.01.

A treatment × day interaction was detected (*P* = 0.04) for exit velocity, which was greater (*P* = 0.03) for CON vs. BAS calves on day 14 and tended (*P* = 0.08) to be greater for CON vs. BAS calves on day 7 (Figure 2). No treatment differences were detected (*P* = 0.91) for chute score (data not shown). A treatment × day interaction was detected (*P* = 0.01) for physical activity, which was greater for CON vs. BAS calves on day 1 (*P* < 0.01), but greater (*P* = 0.01) in BAS vs. CON on day 2 (Figure 2).

**Figure 3.**
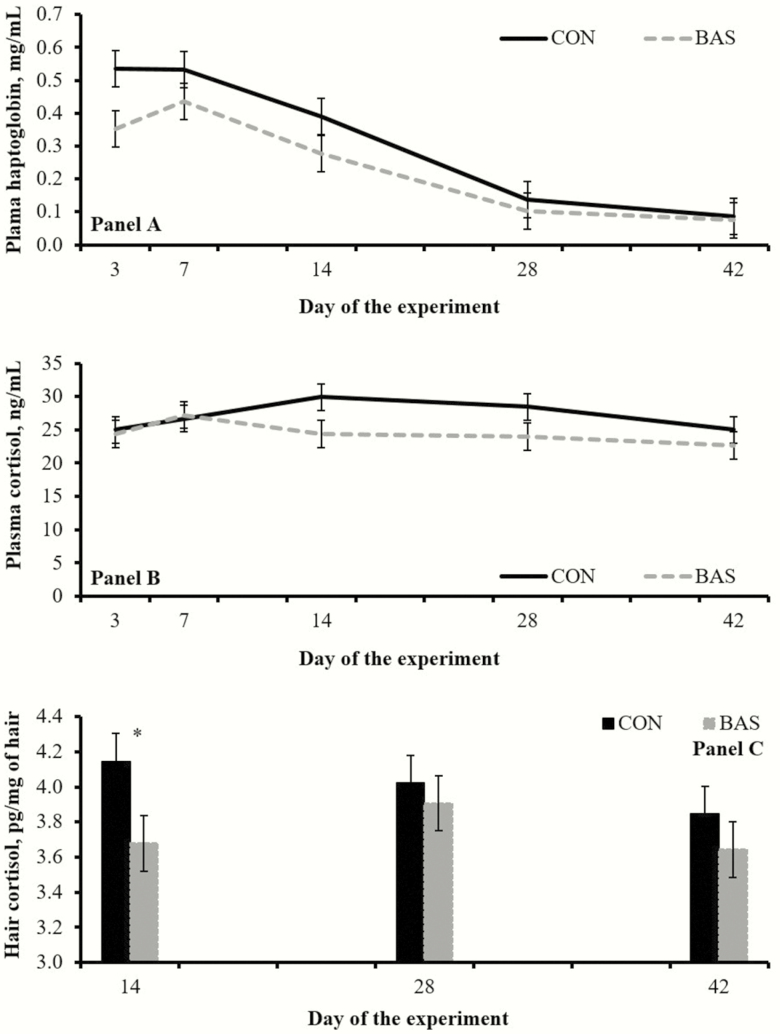
Concentrations of plasma haptoglobin (A) and cortisol in plasma (B) or hair from tail-switch (C) from beef calves receiving (BAS; *n* = 40) or not (CON; *n* = 40) a bovine appeasing substance at weaning (day 0). Values from day 0 were used as independent covariate in each respective analysis. A treatment effect was detected (*P* = 0.03) for plasma haptoglobin (0.248 vs. 0.336 mg/mL for BAS and CON, respectively; SEM = 0.028), and a treatment × day interaction (*P* = 0.03) detected for hair cortisol. No treatment differences were noted (*P* ≥ 0.32) for plasma cortisol concentrations. In (C), *P = 0.05.

**Figure 4.**
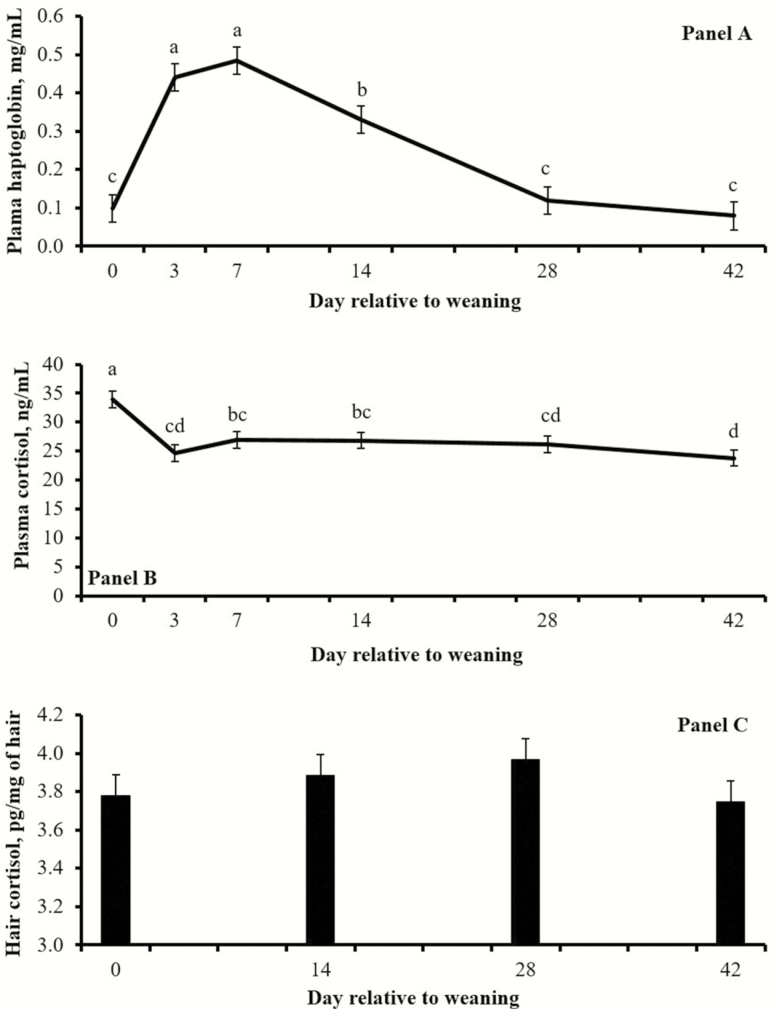
Concentrations of plasma haptoglobin (A) and cortisol in plasma (B) or hair from tail-switch (C) from beef calves weaned on day 0. Day effects were detected (*P* < 0.01) for plasma haptoglobin and plasma cortisol, but not (*P* = 0.23) for hair cortisol concentrations. Days with different superscripts (a,b,c,d) differ (*P* ≤ 0.05).

No treatment effects were detected (*P* ≥ 0.32) for concentrations of plasma cortisol (Figure 3), whereas mean plasma haptoglobin concentrations during the experiment were greater (*P* = 0.03) in CON vs. BAS calves (Figure 3). A treatment × day interaction (*P* = 0.03) was detected for hair cortisol concentrations, which were greater (*P* = 0.05) in CON vs. BAS on day 14, but did not differ (*P* ≥ 0.38) on days 28 and 42 (Figure 3). Day effects were detected (*P* < 0.01) for plasma concentrations of cortisol and haptoglobin, but not (*P* = 0.23) for hair cortisol concentrations (Figure 4).

## DISCUSSION

Weaning is one of the most stressful events within the beef production cycle, the process of which is known to stimulate adrenocortical and inflammatory reactions that impact cattle performance (Cooke, 2017). During weaning, calves are handled for routine processing, creating the opportunity for the application of novel technologies such as BAS. Accordingly, day effects observed for plasma cortisol and haptoglobin corroborate that calves experienced an adrenocortical and subsequent acute-phase protein response elicited by weaning (Figure 4).

Circulating cortisol concentrations have been widely used as a biomarker of stress in cattle (Carroll and Forsberg, 2007); however, BAS administration did not impact cortisol concentrations in weaned calves (Table 1). Handling cattle for blood sampling also elicits an acute stress response that rapidly increases circulating cortisol (Schubach et al., 2017), possibly confounding results. For these reasons, cortisol concentrations in hair from the tail-switch were evaluated. This variable has been recently validated as a biomarker of chronic stress in cattle (Moya et al., 2013), as cortisol is gradually accumulated in the emerging hair and its concentration represents long-term adrenocortical activity (Schubach et al., 2017). Heightened adrenocortical function has also been positively associated with circulating haptoglobin concentrations in cattle (Cooke, 2017). Corroborating this rationale, BAS administration resulted in reduced cortisol concentrations in hair from the tail-switch on day 14 (Figure 4) and overall reduced circulating haptoglobin concentrations (Table 1), supporting our hypothesis that BAS administration alleviates stress elicited by weaning. Moreover, BAS appears to be active for 15 d upon administration (Cooke et al., 2020), when treatment differences on hair cortisol and plasma haptoglobin were more evident in the present experiment (Figure 3).

Calves physically separated from their dams exhibit an increase in physical activity associated with an increase in psychological stress (Solano et al., 2007). Previous research has demonstrated exploration behavior is significantly correlated with habituation, defined as a behavioral response decrement resulting from repeated stimulation (Rodríguez-Prieto et al., 2011). Hence, the decreased physical activity exhibited by BAS calves on day 1 (Figure 2) may be associated with lessened psychological stress response to weaning, whereas their greater physical activity on day 2 may be related to accelerated habituation to their novel environment. Cattle temperament has been directly associated with behavioral stress and subsequent circulating cortisol concentrations (Cooke, 2014), and thus expected to be impacted by BAS. Accordingly, reduced exit velocity of BAS calves on days 7 and 14 (Figure 2) corroborate with treatment differences noted in plasma haptoglobin and hair cortisol concentrations, and further indicates that BAS is active for 15 d upon administration.

Overall ADG, TMR intake, and G:F during the 42-d experimental period were not improved by BAS (Table 1). However, ADG was improved in BAS calves during the initial 28 d after weaning (Table 1), which can be mostly attributed to their increased dry matter intake (DMI) during week 1 (Figure 1) given that no treatment differences were observed in G:F analyzed by week. Stress and its physiological consequences depress DMI in cattle (Cooke, 2017), and the use of BAS probably hastened postweaning TMR consumption by alleviating these outcomes and allowing calves to habituate faster to their new environment. Moreover, performance benefits were noted early in the experiment, when treatment differences in temperament, hair cortisol, and plasma haptoglobin were noticeable. Perhaps additional administrations of BAS, such as every 14 d, were warranted to extend its productive and welfare benefits throughout the 42-d experiment.

## IMPLICATIONS

Administration of BAS upon weaning enhanced calf initial ADG and TMR intake, improved behavioral responses, while reducing hair cortisol and plasma haptoglobin concentrations. Collectively, these outcomes suggest that BAS administration alleviated the stress associated with weaning. Additional research is still warranted to further examine the benefits of BAS in beef production systems, including multiple BAS administrations. Nonetheless, results from this experiment suggest use of BAS as a strategy to improve welfare and productivity of weaned beef calves.
